# Notch3 inhibition enhances sorafenib cytotoxic efficacy by promoting GSK3β phosphorylation and p21 down-regulation in hepatocellular carcinoma

**DOI:** 10.18632/oncotarget.1221

**Published:** 2013-08-24

**Authors:** Catia Giovannini, Michele Baglioni, Marco Baron Toaldo, Cristiano Ventrucci, Stefania D'Adamo, Mario Cipone, Pasquale Chieco, Laura Gramantieri, Luigi Bolondi

**Affiliations:** ^1^ Center for Applied Biomedical Research (CRBA), S.Orsola-Malpighi University Hospital, Bologna, Italy; ^2^ Department of Clinical Medicine University of Bologna, Bologna, Italy; ^3^ Department of Veterinary Medical Science University of Bologna, Bologna, Italy

**Keywords:** ERK, drug resistance, angiogenesis, cell proliferation, Notch3

## Abstract

Sorafenib (Nexavar), a multiple kinase inhibitor, is the only clinically approved drug for patients with advanced HCC. However, its therapeutic success is limited by the emergence of drug resistance. Here we found that p21 and pGSK3β^Ser9^ are major players in the resistance to sorafenib. We recently reported that aberrant Notch3 expression in HCC contributes to doxorubicin resistance *in vitro* and, therefore, we focused on the mechanisms that associate Notch3 to acquired drug resistance. In this study we first found that Notch3 inhibition significantly increased the apoptosis inducing effect of sorafenib in HCC cells via specific down-regulation of p21 and up-regulation of pGSK3β^Ser9^. Using a mouse xenograft model we further found that Notch3 depletion combined with 21 days of sorafenib treatment exerts a substantial antitumor effect *in vivo*. Interestingly, we showed that, upon exposure to sorafenib treatment, Notch3 depleted xenografts maintain lower levels of p21 and higher levels of pGSK3β^Ser9^ than control xenografts. Thus, this study demonstrated that inhibition of Notch3 signaling prevents HCC-mediate drug resistance and sensitizes HCC cells to sorafenib. Finally, we validated our *in vitro* and *in vivo* results in primary human HCCs showing that Notch3 protein expression positively correlated with p21 protein expression and negatively correlated with pGSK3β^Ser9^ expression. In conclusion, the results presented in this study demonstrated that Notch3 silencing enhances the effect of sorafenib by overcoming drug resistance. Notch3 inhibition in combination with sorafenib can be a promising strategy for treatment of HCC.

## INTRODUCTION

Hepatocellular carcinoma (HCC) is a highly malignant liver tumor that most often arises in cirrhotic livers and is quite unrensponsive to common chemotherapeutic agents. In liver cirrhosis, which is today considered a premalignant condition, it is often possible to observe early signs of derangement in the same proliferative and apoptotic pathways driving the growth of HCC cells. Therefore, chemotherapeutic strategies cannot be selective for neoplastic cells and, given the central importance of this organ, liver failure is a deadly complication of HCC therapy. The recent introduction of molecularly targeted drugs has opened a new scenario for HCC treatment. In particular, sorafenib, a multi-kinase inhibitor mainly acting on vascular cells, was shown to significantly improve survival in patients with advanced HCC (SHARP and ASAP), even though prognosis still remained dismal with mean survival rates assessing at 10 and 6.2 months in the different settings (contr sharp and asap) [1, 2]. Thus the exploration of possible combinations between targeted drugs whose effects are addictive or synergic, raises the possibility to find effective combinations with low toxicity. Accordingly, molecules expressed in HCC tissue and not in cirrhosis represent targets of considerable interest to preserve non neoplastic tissue and reduce side effects. The Notch3 receptor is aberrantly expressed in nearly 80% of HCCs with a negligible expression in normal liver as well as in cirrhotic tissue surrounding HCC [3]. Given the fundamental roles played by Notch in cell fate during embryonic development, it is not a surprise that alterations in Notch signaling have been associated with tumor development [4]. Inhibition of Notch expression by antisense retrovirus or pharmacologic blocking of γ-secretase activity has shown striking antineoplastic effects in Notch-expressing transformed cells *in vitro* and in xenograft models [5, 6]. Recently, we showed that Notch3 depleted HCC cells have the same rate of apopotosis of control cells. However, Notch3 silencing in liver cancer cells was able to strongly enhance the therapeutic effects of doxorubicin by up-regulating p53-dependent apoptosis [7]. With regard to HCC resistance to chemotherapeutic agents, we hypothesized that Notch3 may function as a positive factor for multi-drug resistance. Indeed there is evidence that Notch3 over-expression confers resistance to carboplatin and is related to the recurrence of ovarian cancer [8].

The main purposes of the present study are to assess whether Notch3 inhibition enhances sorafenib effects in HCC and to individuate which molecular pathways are interacting in their therapeutic effects.

We showed that the specific block of Notch3 signaling with shRNA enhanced sensitivity to sorafenib *in vitro* and *in vivo*. These data suggest that Notch3 inhibition holds promise as an additional strategy to improve molecular cancer therapy, in particular when resistance to and/or escape from existing therapies evolve.

## RESULTS

### Notch3 KD enhances apoptosis of Sorafenib treated HCC cells

Sorafenib was approved for HCC therapy in 2007 and represents the standard of care for patients with advanced-stage disease [9]. Sorafenib slows tumor growth by blocking different signaling pathways involved in cell proliferation [10] and angiogenesis.

**Figure 1:**
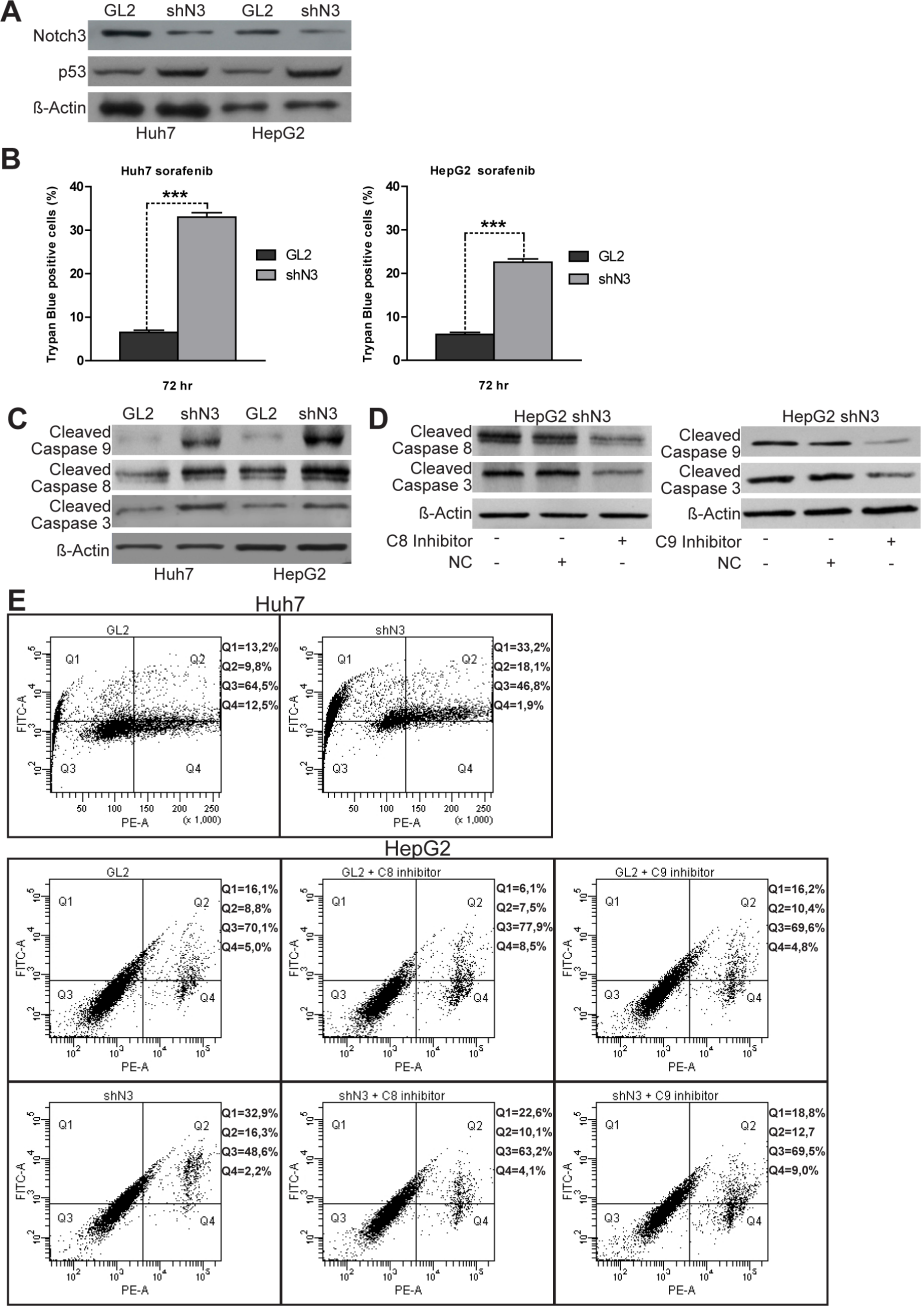
Notch3 KD enhances the proapoptotic effect of sorafenib in vitro (A) Efficacy of Notch3 KD and p53 expression was measured by western blotting in Huh7 and HepG2 cells. (B) The viabilities of control and Notch3 KD Huh7 and HepG2 cells in response to 4 μM of sorafenib were quantified by trypan blue uptake after 72 h of treatment. Results are the mean of three independent experiments (+/− S.E.). P values (by two tailed student's t test) were < 0.001 for shN3 vs GL2 in both the analyzed cell lines. (C) shN3 and GL2 control cells were treated with 4 μM of Sorafenib for 72 h and the levels of Cleaved Caspase 8, 9, 3 and Actin were analyzed by western blotting. (D) Expression levels of Cleaved Caspase 8, 9 and 3 were evaluated in HepG2 Notch3 silenced cells treated with 4μM of sorafenib for 72 h and 10μM of caspase inhibitors or negative control to test inhibitors efficacy. (E) After treatment with 4μM of sorafenib for 72 h HepG2 and Huh7 cells were labeled with annexin V-FITC and propidium iodide. The distribution pattern of live and apoptotic cells was determined by FACS analysis. The same analyses was performed in HepG2 cells treated with 4μM of sorafenib and caspase inhibitors for 72h.Viable cells display no annexin and propidium iodide staining (Q3); early-stage apoptotic cells display high annexin and low propidium iodide staining (Q1); late-stage apoptotic cells display high annexin and high propidium iodide staining (Q2); DNA fragmentation is represented by high propidium iodide and low annexin staining (Q4). X-axis represents propidium staining (PE) and y-axis represents FITC staining. Data are representative of at least three independent experiments. GL2: negative control shRNA; shN3: Notch3 shRNA; C8: caspase 8; C9: caspase 9; NC: negative control (Z-FA-FMK).

We initially explored the effects of Notch3 ablation (Fig. 1A) on sorafenib activity in the two HCC cell lines used in this study. Trypan dye uptake in multiple experiments revealed that after 72 h of sorafenib exposure the mortality due to Notch3 KD increased by 3,8 and 5 fold in HepG2 and Huh7 cells respectively (Fig.1B). We found that sorafenib treatment in Notch3 KD cells resulted in an increase of Caspase-8, Caspase-9 and Caspase-3 cleavage in conjunction with enhance Annexin V-FITC intensity (Fig.1C and 1E) but LDH was not increased in the medium when compared to GL2 cells (Suppl. Fig 1C). Taken together these results indicate that sorafenib treated Notch3 KD cells die because of apoptosis. Next we better examined the contributions of either apoptotic pathways for sorafenib induced apoptosis using Caspase-8 and Caspase-9 selective inhibitors (Fig. 1D). Annexin V-FITC staining showed a reduction in apoptosis in cells treated with caspase 8 inhibitor both in GL2 and in Notch3 depleted cells whereas caspase 9 inhibitor affects apoptosis only in Notch3 silenced cells (Fig. 1E) in line with the high levels of p53 (Fig. 1A) [7]. Taken together our data show that in Notch3 depleted cells sorafenib activates both intrinsic and extrinsic apoptotic pathway.

### Effects of Notch3 depletion and sorafenib treatment on in vivo tumor growth and angiogenesis

Targeting tumor vasculature as a cancer therapy is an established concept to benefit patients with a wide variety of tumor types because tumor angiogenesis implies a rapidly growing tumor.

**Figure 2:**
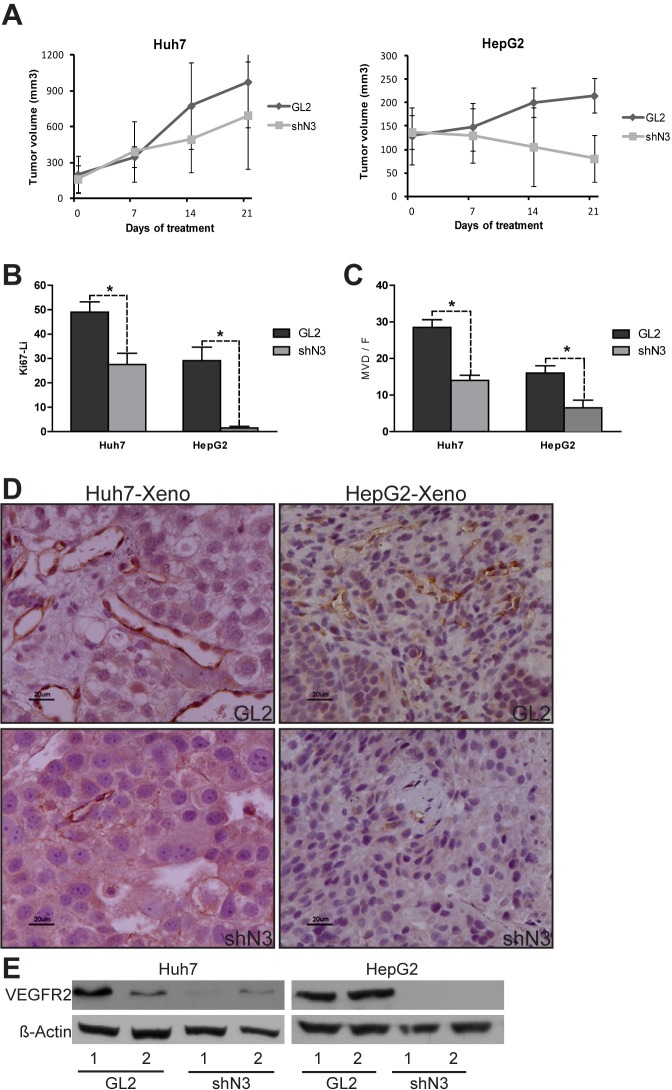
In vivo evidence of the role of Notch3 in sorafenib resistance (A-B) HepG2 and Huh7 cells were subcutaneously injected in the flank of NOD/SCID mice. (A) A difference in tumor growth was evident between GL2 negative control vs shN3 in both the analyzed cell lines. P=0.04 and P=0.01 at t-test for Huh7 and HepG2 respectively after 21 days of Sorafenib treatment. The results are representative data of two independent experiments (+/− S.E). (B) Quantification of growt fraction measured by Ki67 staining. (C) Quantification of tumor angiogenesis measured by immunohistochemical staining of MVD. Proliferation and angiogenesis were quantified as described in the methods. The numbers were the average of counting 7 field in each sample. *, P<0.05 (by two tailed student's t test). (D) Immunohistochemistry of CD31 in representative cases of HepG2 and Huh7 xenografts. Scale bars= 20 μm. (E) Western blot analysis of two representative GL2 xenografts (1-2) and two representative shN3 xenografts (1-2). VEGFR2 was down-regulated in the shN3 xenografts compared to GL2 xenograts. GL2: negative control shRNA; shN3: Notch3 shRNA.

In addition to targeting Raf serine/threonine kinases sorafenib inhibits several RTKs known to promote angiogenesis, as VEGFR2, in human endothelial cells [11]. Interestingly, both VEGFR2 and CD31, a transmembrane molecule expressed on endothelial cells, have been shown to transduce signals that mediate angiogenesis, vascular remodelling and cellular proliferation and their expression correlates with prognosis in HCC [12]. Our experiments showed that the treatment with sorafenib (60 mg/kg/d) for 21 days inhibited tumour growth significantly more in both Huh7 (p=0.04) and HepG2 (p=0.01) Notch3 depleted xenografts than in GL2 xenografts. (Fig.2A). Indeed, after 21 days of sorafenib exposure Notch3 depleted xenografts showed a much lower proliferation and vessel density than Notch3 undepleted ones, as shown by immuno-staining for Ki67, VEGFR2 and CD31 (Fig.2B-E).

### Notch3 and Sorafenib share common pathways

**Figure 3:**
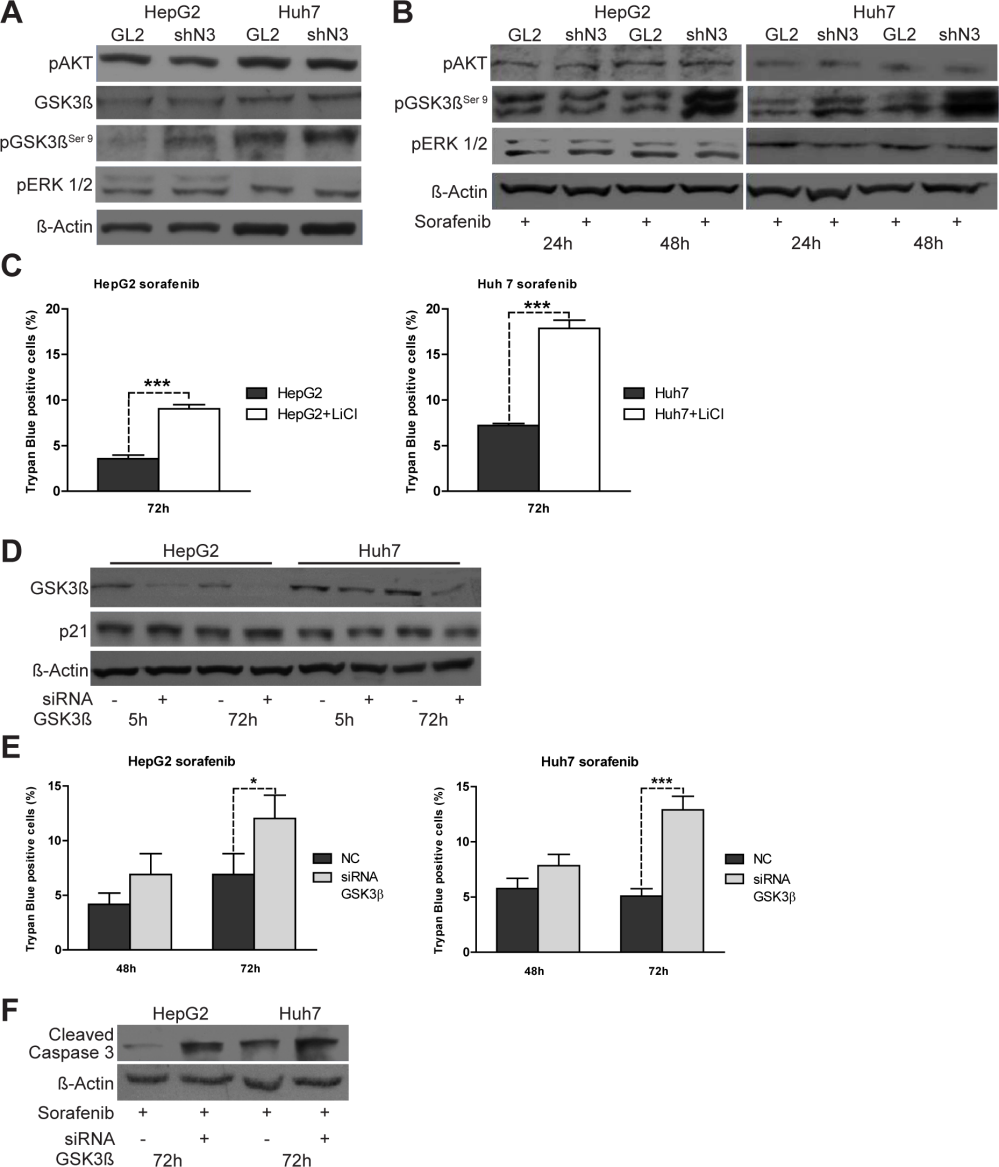
pGSK3βser9 expression is regulated by Notch3 and enhances the effect of sorafenib (A) pAkt, pERK, total-GSK3β and pGSK3β^ser9^ protein levels in control and Notch3 KD cells were analyzed by western blotting. (B) The same proteins analyzed in (A) were evaluated after exposure to sorafenib for 24 and 48 h. GL2: negative control shRNA; shN3; Notch3 shRNA. (C) HepG2 and Huh7 cells were treated with 20 mM of LiCl. Cells were treated 5 h post lithium chloride treatment with 4 μM of sorafenib for 72 h and cell death was assessed by trypan blue uptake. Results are shown as the means of three independent experiments (+/− S.E.). P values (by two tailed student's t test) were p<0.001 for HepG2 and Huh7 VS LiCl treated cells. (D) HepG2 and Huh7 cells were transfected with GSK3β siRNA or scrambled RNA and GK3β knockdown and p21 protein levels were evaluated 5 h and 72 h post-transfection by western blot. (E) Cell viability by trypan blue uptake was scored three times as a function of 4 μM of sorafenib treatment for the indicated time. Sorafenib treatment was started five hours post siRNA transfections. Results are shown as the means of three independent experiments (+/− S.E.). **, P<0.01 (by two tailed student's t test). (F) Cells transfected with GK3β siRNA or scrambled RNA were treated with 4 μM of Sorafenib for 72 h and the levels of cleaved caspase 3 were analyzed by western blotting.

To better investigate the mechanisms associated with the enhanced sorafenib sensitivity observed in Notch3 depleted cells, we first examined the phosphorylation status of well established targets of sorafenib such as ERK1/2 and Akt [13] in Notch3 KD cells. As shown in Figures 3A & 3B, GL2 and Notch3 KD cells had very similar expression of p-ERK1/2 and p-Akt in either untreated or sorafenib treated cells, suggesting that these molecules are not associated with the response to sorafenib in Notch3 KD cells. We next examined GSK3β, a kinase activated by sorafenib, and its inhibitory phosphorylation status (pGSK3β^Ser9^). We found that pGSK3β^Ser9^ was markedly increased in Notch3 KD cells compared with GL2 cells (Fig.3A) and that the high pGSK3β^Ser9^ levels were maintained in response to sorafenib exposure (Fig.3B).

On this regard we further found that the pharmacological or molecular inhibition of GSK3β, by lithium treatment or siRNA transfection respectively, increased sorafenib-induced apoptosis in both HCC cell lines (Fig.3C, E-F). Therefore, GSK3β activity seems to protect from sorafenib toxicity and the high level of the inactive pGSK3^Ser9^ form found in Notch3 KD cells could play a critical role in the sorafenib increased lethality in these cells.

Finally, we examined whether sorafenib treatment modifies the expression of two proteins regulated by Notch3, p21 and p53, known to affect the responsiveness to chemotherapeutics drugs [7, 14]. Supplemental Figure 2A shows that the levels of p21 and p53 proteins were reduced in response to sorafenib exposure between 24 and 48 hours. On the mRNA side, semi-quantitative RT-PCR analyses in sorafenib treated cells revealed a decreased p21 mRNA expression but an increased expression of p53 mRNA (Suppl. Fig.2B). Interestingly, Notch3 depleted cells retain lower levels of p21 and the same levels of p53 then GL2 cells after exposure to sorafenib (Suppl. Fig.2C).

### P21 down-regulation contributes to sorafenib-induced cell death in HCC cells

**Figure 4:**
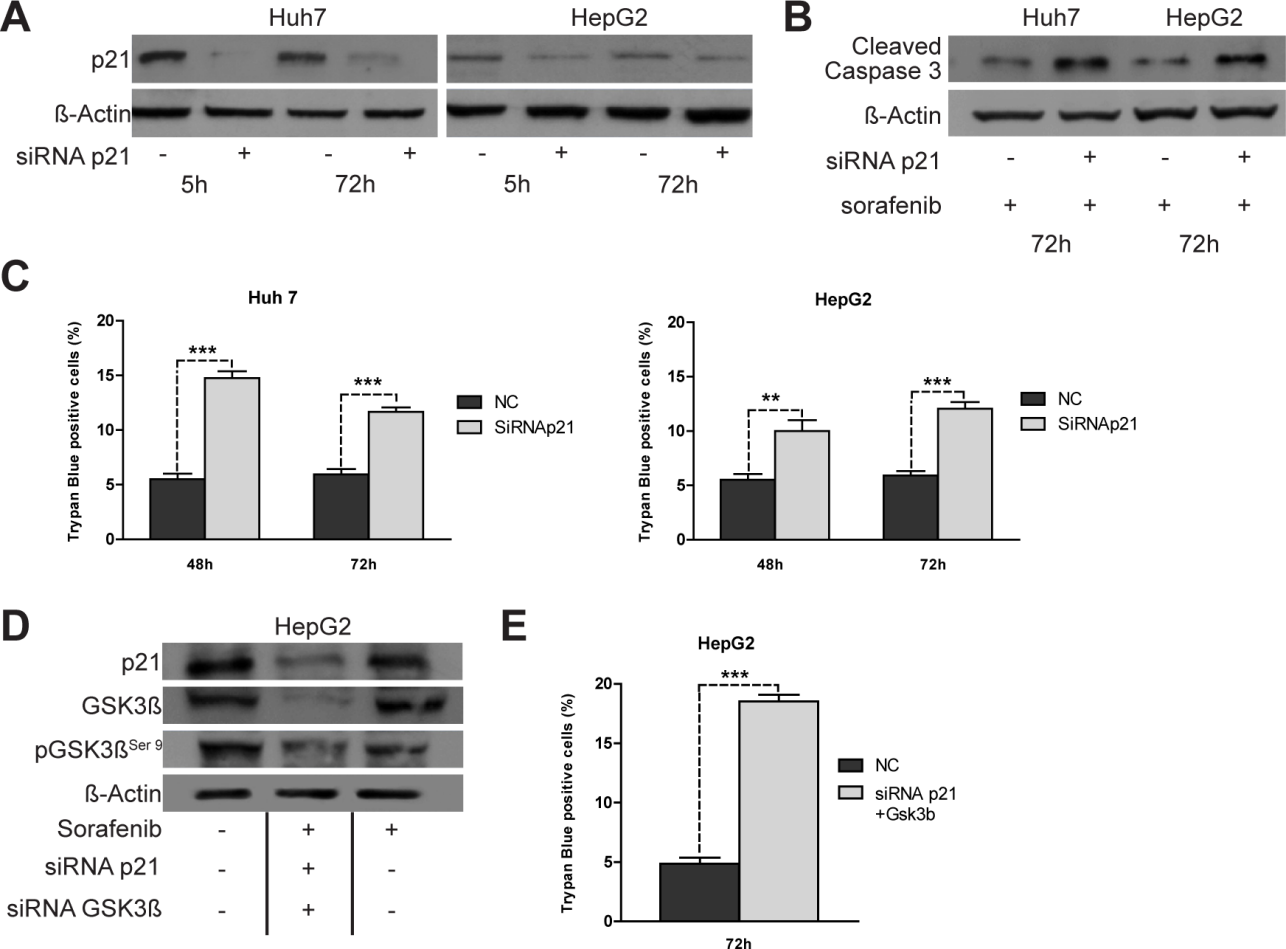
Effects of knockdown of p21 expression on apoptosis induced by sorafenib (A) Huh7 and HepG2 were transiently transfected with a pool of siRNAs directed against p21 or negative control (NC) for 5 h and 72 h. The level of p21 protein was evaluated by western blot.(B) Five hours post-transfection cells were treated with 4 μM of Sorafenib for 72 h and the levels of cleaved caspase 3 were analyzed by western blotting. (C) The effect of p21 silencing on sorafenib (4 μM) induced apoptosis were assessed by trypan blue uptake as the means of three independent experiments (+/− S.E.). **, P<0.01 (by two tailed student's t test). (D) HepG2 cells were simultaneously transfected with p21 and GSK3β siRNA. 5 h post-transfection cells were treated with 4 μM of Sorafenib for 72 h and the levels of p21, GSK3β and pGSK3β^ser9^ were analyzed by western blotting. (E) Cell viability by trypan blue uptake was scored three times as a function of 4 μM of sorafenib treatment for 72 h. Results are shown as the means of three independent experiments (+/− S.E.). ***, P<0.001 (by two tailed student's t test).

To determine if lower p21 levels were functionally associated with the enhanced sorafenib sensitivity shown by Notch3 KD cells, we ablated endogenous p21 expression by transient siRNA transfection (Fig.4A). Transfected cells were treated with 4 uM sorafenib for 48 h and 72 h. p21 knockdown increased apoptosis induced by sorafenib as revealed by trypan blue uptake in three independent experiments and by increased levels of cleaved caspase 3 (Fig.4B-C). From the data it appears, that sorafenib treatment was more effective in Notch3 KD cells than in p21 silenced cells, suggesting that p21 was not the only reason for the enhanced sorafenib sensitivity following Notch3 depletion. In addition to transcriptional regulation by both p53 and Hes1 proteins, p21 functions can be regulated at post-translational level by GSK3β [15]. However, the lower levels of p21 observed in sorafenib treated HepG2 and Huh7 were not dependent by GSK3β. Indeed GSK3β silencing did not down-regulate p21 protein expression (Fig.3D) suggesting that the effect of sorafenib in p21 depleted cells was independent of GSK3β.

### Notch3 enhanced apoptosis of sorafenib treated cells depends on p21 and pGSK3βSer9

To determine whether p21 and pGSK3β^Ser9^ are major factors associated with the enhanced sorafenib sensitivity in Notch3 KD cells, we simultaneously ablated endogenous levels of both p21 and GSK3β by transient siRNA transfection of HepG2 cells (Fig.4D). Five hours post-transfection cells were treated with 4 μM of sorafenib for 72 h. Down regulation of both p21 and GSK3β resulted in enhanced cell death as revealed by trypan blue uptake in three independent experiments (Fig.4E). Cell death upon sorafenib exposure increased 3.85 fold in cells silenced for both p21 and GSK3β compared to negative control (Fig.4E) (p<0.001). Considering that Notch3 depletion alone enhances sorafenib sensitivity 3.8 fold in HepG2 cells (Fig.1B), it seems that p21 and pGSK3β^Ser9^ expression are the main reasons for the increased sorafenib sensitivity of Notch3 silenced cells in vitro.

### P53 is not required to enhance the cytotoxicity of sorafenib

To determine if lower p53 levels observed in sorafenib treated HepG2 and Huh7 cells were functionally associated with drug sensitivity, we ablated endogenous p53 expression by transient siRNA transfection (Suppl.Fig.3A). Transfected cells were treated with sorafenib for 48 h and 72 h. Down-regulation of p53 reduced cellular death of both HepG2 and Huh7 only after 48 h of sorafenib exposure, as revealed by trypan blue uptake. After 72 h of treatment with sorafenib no difference were observed in cell death between control and p53 silenced cells (Suppl. Fig.3B) probably because sorafenib treatment down-regulates p53 (Suppl. Fig.2B) causing the same effect of p53 silencing. Our findings are in line with a recent study showing that the sensitization effect of sorafenib on cisplatin can be mediated via both p53-dependent and-independent mechanisms [16]. These results strengthen the above observation indicating that p21 and pGSK3β^Ser9^ are the major effectors of the enhanced sorafenib sensitivity of Notch3 depleted HepG2 and Huh7 cells.

### Sorafenib regulates p21 and pGSK3βSer9through ERK dependent and independent mechanisms

ERK proteins associates with and phosphorylates different proteins including GSK3β and p21 resulting respectively in their inactivation or degradation [17, 18]. In addition ERK1/2 act as activators of p53 and consequent cellular response [19]. To analyze if ERK1/2 are responsible for the alterations observed in the levels of p21 and pGSK3β^ser9^ following exposure to sorafenib, their expression was assessed in ERK1/2 silenced cells.

**Figure 5:**
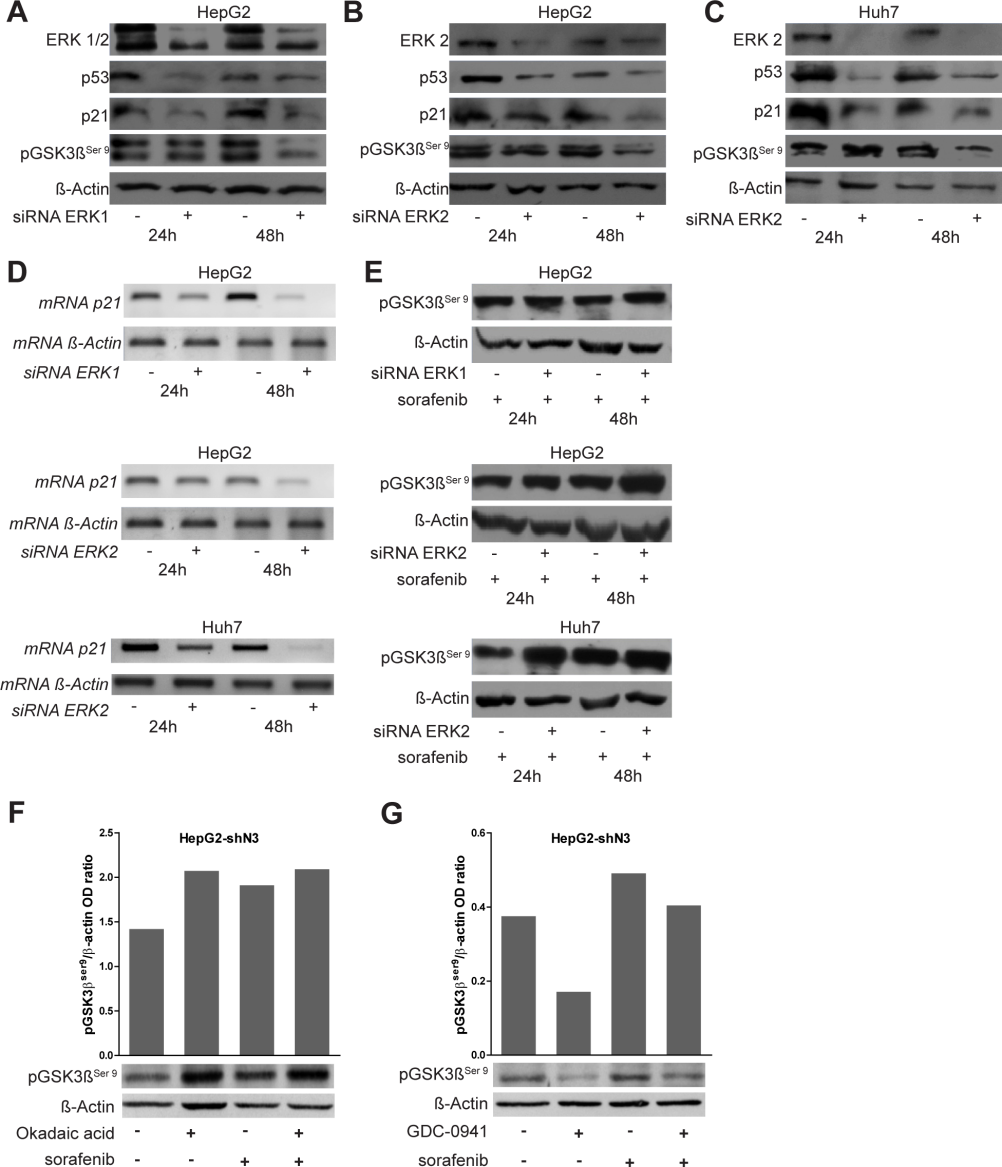
Role of ERK1/2 on p53, p21 and pGSK3βser9 regulation (A-B-C) HepG2 and Huh7 cells were transfected with a pool of ERK1 and ERK2 siRNAs or scrambled RNA and ERK1/2 knockdown, p21, p53 and pGSK3β^ser9^ protein levels were evaluated 24 h and 48 h post-transfection by western blot. ERK1/2 silencing resulted in reduced levels of all the analyzed proteins. (D) Semi-quantitative RT-PCR expression analysis of p21 in ERK1/2 silenced cells. (E) pGSK3β^ser9^ protein levels were analyzed after exposure to 4 μM of sorafenib for 24 and 48 h in ERK1/2 silenced cells by western blotting. (F-G) pGSK3β^ser9^ protein levels were evaluated 12h post treatments with 60nM of Okadaic acid or 150nM of GDC-0941 or 4 μM of sorafenib or combined treatments by western blot. The levels of proteins showed in panel F and G were quantified and expressed as a ratio with respect to β-actin control.

ERK1/2 silencing resulted in decreased pGSK3β^Ser9^, p21 and p53 protein levels (Fig.5A-C). Semi-quantitative RT-PCR analyses revealed similar results for p21 gene transcription (Fig.5D). These results lets us to hypothesize that ERK1/2 mediate p21 down-regulation via p53. However, sorafenib treatment of ERK1/2 silenced cells resulted in increased pGSK3β^Ser9^ (Fig. 5E) suggesting that sorafenib regulates GSK3β phosphorylation through mechanisms independent of ERK1/2. Accordingly, pGSK3β^Ser9^ was up-regulated in both untreated and sorafenib treated Notch3 KD cells although pERK1/2 protein levels were unchanged (Fig.3A-B). To further investigate the mechanism involved in GSK3β phosphorylation, HepG2 Notch3 silenced cells were treated with a phosphate inhibitor (Okadaic acid) ore with a kinase inhibitor (GDC-0941) alone or in combination with sorafenib. Phosphatase inhibition resulted in the same increase in pGSK3β^Ser9^ observed in sorafenib treated cells. Interestingly combined treatment has the same effect on GSK3β phosphorylation than single compounds suggesting that this phenomenon is due to phosphatase inactivation by sorafenib (Fig. 5F). Moreover, in agreement with this observation, kinase inhibitor GDC-0941, alone or in combination with sorafenib, reduces the levels of pGSK3β^Ser9^ compared to sorafenib treated cells (Fig. 5G).

### Liver tumor cells confere resistance to sorafenib through pERK1/2 and p21 up-regulation and pGSK3βSer9 downregulation

**Figure 6:**
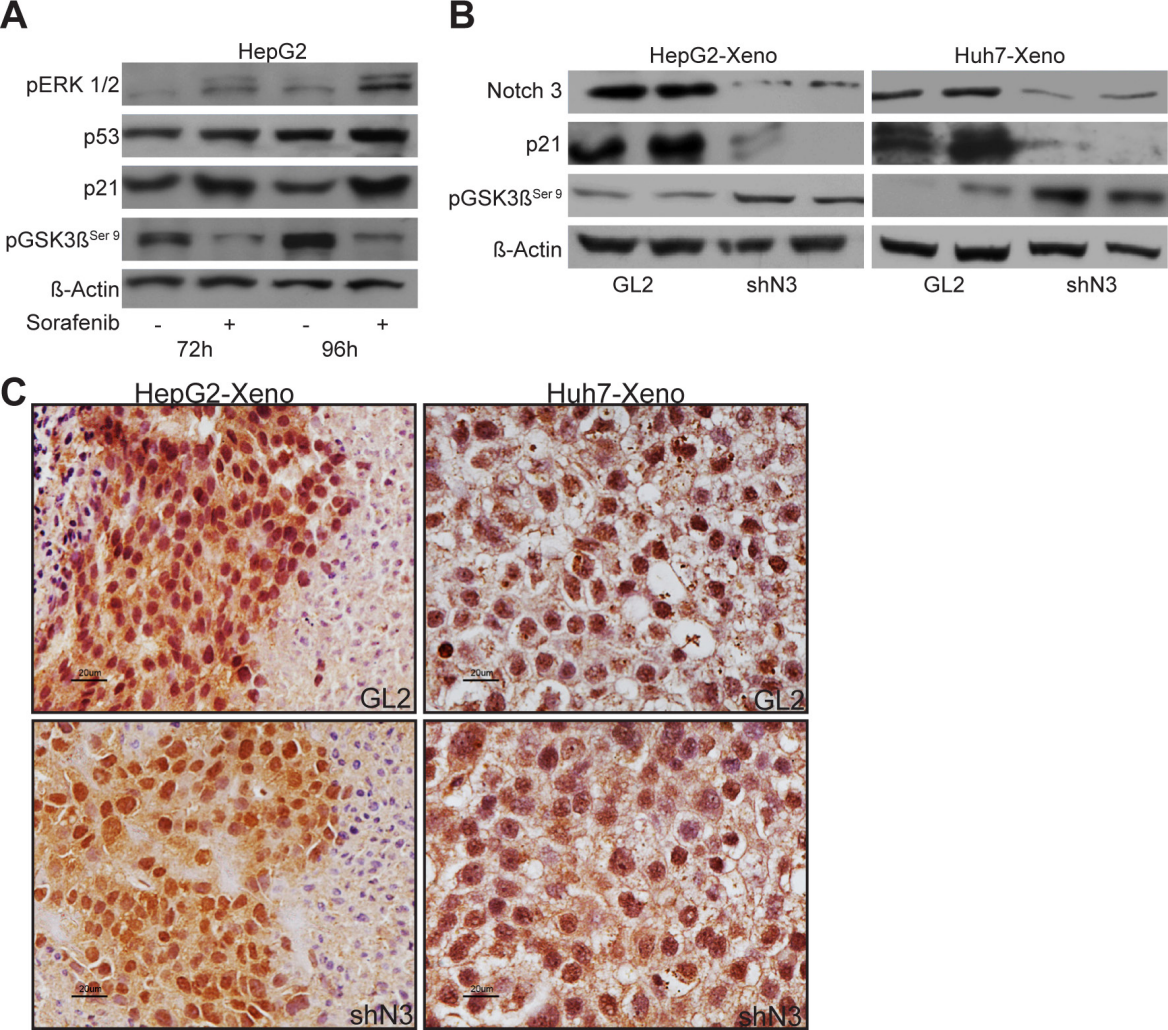
Sorafenib resistance evades blockade of ERK signaling (A) pERK1/2, p53, p21 and pGSK3β^ser9^ protein levels were analyzed after exposure to 4 μM of sorafenib for 72 h and 96 h in HepG2 cells by western blotting. (B) Western blot analysis of Notch3, p21 and pGSK3β^ser9^, expression in HepG2 and Huh7 xenografts evaluated after 21 days from the beginning of the treatment with 60 mg/kg of sorafenib. (C) Immunohistochemistry of pERK1/2 in representative cases of HepG2 and Huh7 xenografts. pERK1/2 accumulation was evident in the nucleus and in the cytoplasm. Scale bars= 20 μm. GL2: negative control shRNA; shN3; Notch3 shRNA.

It was recently shown that ERK phosphorylation levels in tumor tissues correlate with the time to progression of patients affected by HCC. Indeed, in resistant HCC patients ERK1/2 activity was highly increased after 21 days of treatment with sorafenib [20]. We then explored possible mechanisms of sorafenib resistance in the HepG2 cells because an enhanced sorafenib sensitivity due to Notch3 ablation was higher in Huh7 cells than in HepG2 cells *in vitro* (Fig.1B). We found that ERK phosphorylation decreased after 24-48 h of treatment with sorafenib (Suppl.Fig.2A) and gradually increased after long sorafenib exposure (Fig.6A). In line with these observations, increased levels of p21 and decreased levels of pGSK3β^Ser9^ were also observed while p53 protein levels were almost unaffected (Fig.6A).

### In vivo results

Because p21 and pGSK3β^Ser9^ contribute to Sorafenib resistance *in vitro* and since p21 levels and pGSK3β^Ser9^ are down-regulated and up-regulated respectively in Notch3 KD cells, we examined their expression in tumor xenografts after sorafenib treatment. We found that Notch3 KD tumors had significant lower levels of p21 and increased levels of pGSK3β^Ser9^ than GL2 tumors (Fig.6C). In line with the above reported *in vitro* results, Notch3 depletion did not affect phosphorylation and localization of ERK1/2 which resulted almost identical in GL2 and Notch3 tumors. (Fig.6D). The correlation between *in vivo* and *in vitro* studies suggest that, by keeping lower levels of p21 and higher levels of pGSK3β^Ser9^, Notch3 depleted cells did not acquire resistance to sorafenib.

### Notch3 regulates *pGSK3βSer9* and p21 protein levels in human HCC

**Figure 7:**
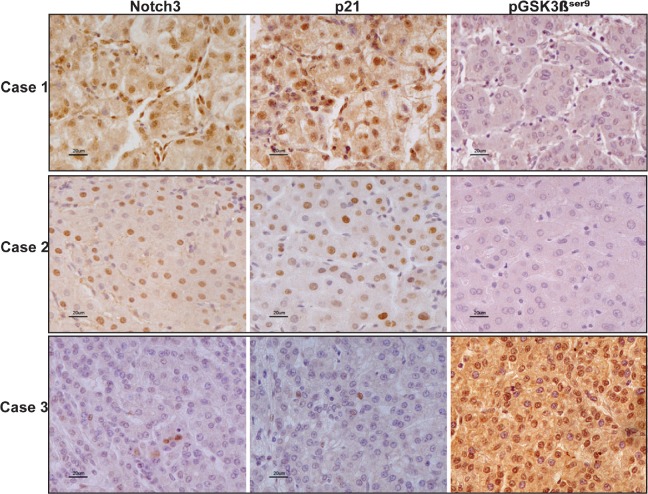
Expression profile of Notch3, p21 and pGSK3βser9 in human HCC Immunohistochemistry analysis in three representative cases showing Notch3, p21 and pGSK3β^ser9^ expression in the same area. Scale bars= 20 μm. Case 1 and Case 2 show Notch3 and p21 nuclear localization whereas they stain negative for pGSK3β^ser9^. Case 3: HCC tissue with Notch3 and p21 negative hepatocytes results positive for pGSK3β^ser9^expression.

To assess to what extent our in-vitro findings are representative of what occurs in human HCC, we analyzed the expression of Notch3, p21 and pGSK3β^Ser9^ proteins in 20 surgically resected HCCs by immunohistochemistry. We found a significant inverse correlation between Notch3 and pGSK3β^Ser9^ proteins accumulation (Spearman ρ= −0.666, p<0.01) and a significant direct correlation between Notch3 and p21 proteins expression (Spearman ρ= 0.681, p <0.01) (Fig.7) suggesting that Notch3 participates in the control of GSK3β phosphorylation and p21 expression in human hepatocellular carcinoma.

## DISCUSSION

It was recently shown, in large clinical trials, that sorafenib treatment significantly prolongs survival of patients with advanced HCC [1, 2], but the tumor response rate was short because of drug resistance [21]. Therefore, it is of great importance to either identify metabolic or signalling cellular pathways that could be targeted to enhance HCC sensitivity to sorafenib, or to understand the mechanisms of sorafenib resistance. Notch pathway is frequently deregulated in human cancer and inhibition of Notch signaling has been described as a promising strategy for tumors treatment [22]. It functions as an oncogene but can also act as a tumour suppressor. These apparently contradictory functions of Notch signalling strongly indicate that the effects of Notch activation are dependent on the cellular context [23]. In the present study we report that Notch3 depletion enhances sorafenib toxicity towards HCC cells both *in vitro* and *in vivo*. We found that either sorafenib treatment or Notch3 depletion resulted in p21 down-regulation and pGSK3β^Ser9^ up-regulation whereas sorafenib, but not Notch3 depletion, decreased p53 expression. Extensive studies have demonstrated that sorafenib mediates apoptosis through MEK/ERK-dependent and independent mechanisms. Examples include Gadd45β, the translation initiator elF4E and Mcl-1 [24-26]. In line with this we showed that the reduced levels of p21 observed in sorafenib treated cells are ERK1/2 dependent. Conversely, sorafenib can increase pGSK3β^Ser9^ levels whereas ERK1/2 silencing cannot. We then demonstrated that p21 silencing in association with GSK3β silencing or inactivation enhanced sorafenib sensitivity of HepG2 and Huh7 cell lines. Thus our observations are consistent with the active role assigned to p21 in inhibiting apoptosis [20, 27, 28] and with the critical role played by GSK3β in cell survival [29]. As far as p53 is concerned, we found that sorafenib treatment in HCC cells resulted in enhanced mRNA expression but reduced protein levels. The induction of p53 mRNA is probably related to the pro-apoptotic stimulus induced by sorafenib. However, the lower level of p53 protein in sorafenib treated cells were consistent with reduced expression of the sorafenib targets ERK1/2. In support of the latter possibility, siRNA mediated ERK1/2 knockdown also reduced p53 expression in HepG2 and Huh7 cells. Moreover p53 depletion does not affect the sensitivity to long sorafenib exposure in HCC cells suggesting that p53 is not a key factor in mediating sorafenib response. Of interest here is our previous finding that Notch3 protects HCC from doxorubicin-induced death by controlling p53 levels [7]. Therefore, it seems that different pathways might be involved when Notch3 depletion is associated to the administration of a chemotherapeutic agents, like doxorubicin, or to the administration of a biologically active agent [29].

Overall, the above data indicate that the enhanced sorafenib sensitivity observed in Notch3 depleted HepG2 cells was mostly dependent on p21 and pGSK3.

Considering that resistance to sorafenib has been observed in different clinical trials we analyze whether p21 and pGSK3β^Ser9^ were involved in mediating resistance to the drug. ERK1/2 phosphorylation was rapidly reduced after exposure to sorafenib but progressively returned to baseline levels restoring the expression of p21, p53 and pGSK3β^Ser9^ proteins in HepG2 cells. In contrast with this *in vitro* observation, Notch3 KD xenografts expressed lower levels of p21 and higher levels of pGSK3β^Ser9^ than GL2 xenografts upon exposure to 21 days of sorafenib whereas no difference were observed in p53 expression between the two xenografts. *In vivo*, the association of sorafenib and Notch3 depletion resulted in slower tumor growth as demonstrated by reduced tumor volume and decreased Ki-67 labeled growth fraction

Inhibition of tumor angiogenesis is considered a major mechanisms of HCC response to sorafenib. HCC is a hypervascular tumor, and the use of anti-angiogenic therapy has been extensively studied [30]. However anti-angiogenic therapy elicits forms of adaptive resistance that take to treatment failure [31]. Notch signaling participates in multiple aspects of vascular development including angiogenesis, differentiation and remodelling of vascular smooth muscle cells (VSMC) [32]. We proved that combination of sorafenib and Notch3 depletion significantly decreased both CD31 staining and VEGFR2 *in vivo* suggesting that Notch3 signaling by cancer cells plays a role in neo-angiogenesis.

The results presented in this study demonstrate, for the first time, that Notch3 inhibition enhances the effect of sorafenib in human HCC. Importantly, we present evidence that the effects of Notch3 depletion in sorafenib response are mediated by p21 and pGSK3β^Ser9^ and, probably, by neo-angiogenesis inhibition. Our data suggest also that the synergistic activity of sorafenib with Notch3 mainly ends up in avoiding drug resistance. Altough γ-secretase inhibitors are today evaluated as promising inhibitors of Notch signaling in neoplastic disease [33, 34], targeting Notch3 should be preferred for HCC therapy to avoid additional damage to non neoplastic cirrhotic liver [3].

## MATERIAL AND METHODS

### Ethics Statement

Investigation has been conduced in accordance with the ethical standards and according to the Declaration of Helsenki and according to national and international guidelines and has been approved by the authors institutional reviewed board.

### Cell lines and Notch3 knockdown by retroviral transduction of shRNAs

The human hepatocarcinoma cell lines HepG2 and Huh7 were obtained from American Type Culture Collection (ATCC, Rockville, MD, USA) at the end of 2011. Cells have not been authenticated by the authors. HepG2 cells were maintained in Eagle's Minimum Essential Media (MEM) while Huh7 cells were maintained in RPMI. Media were supplemented with 10% of fetal bovine serum (FBS), 100 U/ml of penicillin, and 100 mg/ml of streptomycin (all reagent from ATCC) at 37 °C in 5% CO_2_ incubator. Notch3 knock down (KD) was obtained using short hairpin oligonucleotides targeted to different Notch3 exons inserted into the pSuper.puro expression vector (OligoEngine, Seattle, WA) as previously described (7). Since two Notch3 specific shRNAs were equally effective in our previous study (7) we performed the experiments by selecting a single shRNA (shN3). Cells harbouring a pSuper.puro provirus expressing a GL2 luciferase specific shRNA were used as negative control (35).

### Compounds and cell death assays

Sorafenib was obtained by Bayer Healthcare (BAY 43-9006, Italy), Okadaic acid was purchased from Sigma and GDC-0941 was obtained by Selleckem (Houston, TX, USA). Caspase 8 inhibithor (Z-IETD-FMK), Caspase 9 inhibitor (Z-LEHD-FMK) and negative control (Z-FA-FMK) were from BD (Oxford, England).

Stably infected cell populations of HepG2 and Huh7 were seeded into 6-well dishes and allowed to attach for 24 hours before treatments. Cell death was revealed and quantified by multiple criteria. Our primary quantitative assay was trypan blue uptake. Apoptosis was also revealed by Annexin V-FITC (Bender Medsystems, Vienna, Austria) staining via FACS. Initiation of cell death was assessed by the appearance of cleaved caspase 8, 9 and 3 in western blots (36). Cellular necrosis was assessed by LDH (lactate dehydrogenase) release by control vs. sorafenib treated cells.

### Xenografts mouse model and treatments

Seven-to eight week-old NOD/SCID female mice (Harlan, Udine, Italy) were inoculated subcutaneously with 5 × 10^6^ HepG2 cells (GL2 or shN3) or with 3 × 10^6^ Huh7 cells (GL2 or shN3). Treatment with sorafenib was initiated when tumor nodules reached 130 mm^3^ to 200 mm^3^ in volume as determined by ultrasonography (US) imaging. Sorafenib was given by oral gavages for 21 consecutive days at 60 mg/kg dose; tumor volume during treatment period was monitored weekly by using US imaging. At the end of the treatment, animals were sacrificed and each tumor mass was collected for laboratory analysis.

### Small interfering RNA transfections

HepG2 and Huh7 cells were seeded into 6 well plates and transfected with 20 nM of p53 (Invitrogen), p21(IDT, Thief River Falls, MN, USA), GSK3, ERK1/2 (Santa Cruz Biotechnology) siRNAs or scrambled siRNA (scRNA) using Lipofectamine2000 (InVitogen). Transfection efficiencies were greater than 90% as determined by co-transfection with a fluorescein-labelled siRNA (InVitrogen). Five hours were allowed to elapse before treatment with sorafenib 4 μM. Cells were collected at 5 h and 72 h post-transfection and proteins expression was analyzed by western-blot.

### SDS-PAGE and Western blot analysis

Protein extraction and quantification were performed as previously described [7]. Primary antibodies were as follows: anti-Notch3 polyclonal antibody (sc-5593, Santa Cruz Biotechnology), anti-p21 monoclonal antibody (Clone SX118, Dako, Denmark), anti-p53 monoclonal antibody (Clone DO-7, Dako), anti-Cleaved Caspase 3 monoclonal antibody (9664, Cell Signaling Technology, Beverly, MA), anti-p-ERK monoclonal antibody (sc-7383, Santa Cruz Biotechnology), anti-ERK1/2 polyclonal antibody (9102, Cell Signaling) anti-GSK3β monoclonal antibody (sc-7291, Santa Cruz Biotechnology), anti-p GSK3β monoclonal antibody (9323, Cell Signaling) anti-pAkt monoclonal antibody (2965, Cell Signaling), anti-VEGF Receptor 2 monoclonal antibody (2479, Cell Signaling), anti-CD31 polyclonal antibody (ab28364 Abcam, Cambridge, UK) and anti-β-actin monoclonal antibody (Clone AC-40, Sigma) Immunoreactivites were revealed with the EnVision dextran polymer visualization system (Dako).

### RNA analysis

Total cellular RNAs were prepared with Trizol (InVitrogen, Paisley, Scotland) according to the manufacturer's instructions. Two micrograms of total RNA were reverse-transcribed using Superscript II (InVitrogen). Relative gene expressions were determined by semi-quantitative end-point PCR. PCR primers were as follow:

NOXA (FW 5'cgagaattcgagatgcctgggaag-3',
REV 5' cttggtaccggttcctgagcag-3')
bax (FW 5'acagggtttcaccaggatc-3',
REV 5'- gctgccacccgcaagaagac-3'),
p21 (FW 5' aagaccatgtggacctgtca,
REV 5' ggcttcctcttggagaagat),
p53 (FW 5' gacccaggtccagatgaagct,
REV 5' accgtagctgccctggtaggt),
β ACTIN (FW 5'- gaggcactcttccagccttc-3',
REV 5'- ggatgtccacgtcacacttc-3').

### Patient samples

Twenty patients of both sexes undergoing partial hepatectomy for HCC entered the study. Informed consent was obtained from each patient according to Italian guidelines and the latest version of the Helsinki Declaration. Exclusion criteria were a previous history of local or systemic treatments for HCC. Tissues sample were fixed in 10% formalin and paraffin-embedded for histopathology and immunohistochemistry.

### Immunohistochemistry (IHC)

The presence and localization of pERK1/2, CD31and Ki67 (Dako) in tumor xenografts and the expression of Notch3, p21, pGSK3β^Ser9^, in human HCCs, were immunohistochemically assessed on formalin-fixed, paraffin-embedded sections. Serial 4 μm thick sections were processed for haematoxylin and eosin staining and for immunohistochemistry. Endogenous peroxidases were inhibited by incubating slides in 3% H_2_O_2_–methanol for 20 min at 4°C. For antigen retrieval, slides were immersed in pH 6.0 citrate buffer (pH 6.0) and boiled using a microwave owen. Negative controls were obtained by omitting the primary antibody. Immunoreactivity was revealed with the EnVision system (DAKO), and diaminobenzidine (DAB) as chromogen (Sigma, ST Louis, USA).

Slides were counterstained in Meyer's hematoxylin, coverslipped and examined by light microscopy. Microvessel density (MVD), calculated as the average of microvessel counts in seven randomly selected 40x fields using a motorized stage (Marzhauser, Wetzlar, Germany), was used to represent tumor angiogenesis activity. Ki67 staining was quantified by image cytometry using Image J software (NIH, Bethesda, USA) on at least 7 randomly selected consecutive fields at 40 x and expressed as the percentage of positive nuclei over the total nuclei (Labeling index:LI). Staining was assessed by two independent observers (L. G., C. G.) who evaluated the percentage of positive hepatocytes in each fields. Results represent the average of the percentage from 10 consecutive 20 x magnification fields.

### Statistical analysis

Differences between groups were analyzed using a double-sided Student t-test. Experimental data are expressed as the mean ± SE from three independent experiments. T-test was also used to explore significant difference in tumor growth between control and Notch3 silenced xenografts. Spearman's correlation was used to explore the relationships between Notch3 and p21 or Notch3 and pGSK3β^ser9^ expression in HCC tissues. P-values less than 0.05 were considered statistically significant. Statistical analyses were performed using SPSS version 19.0.

## Supplemental Figures


