# Supplementary material for: Notch3 inhibition enhances sorafenib cytotoxic efficacy by promoting GSK3β phosphorylation and p21 down-regulation in hepatocellular carcinoma
Source: Oncotarget. 2013 Aug 24;4(10):1618–31. doi: 10.18632/oncotarget.1221 (PMC3858550; doi:10.18632/oncotarget.1221)
Supplement: Supplementary file 1 [file oncotarget-04-1618-s001.pdf]

Notch3 inhibition enhances sorafenib cytotoxic efficacy by promoting GSK3β phosphorylation and p21 down-regulation in hepatocellular carcinoma – Giovannini et al

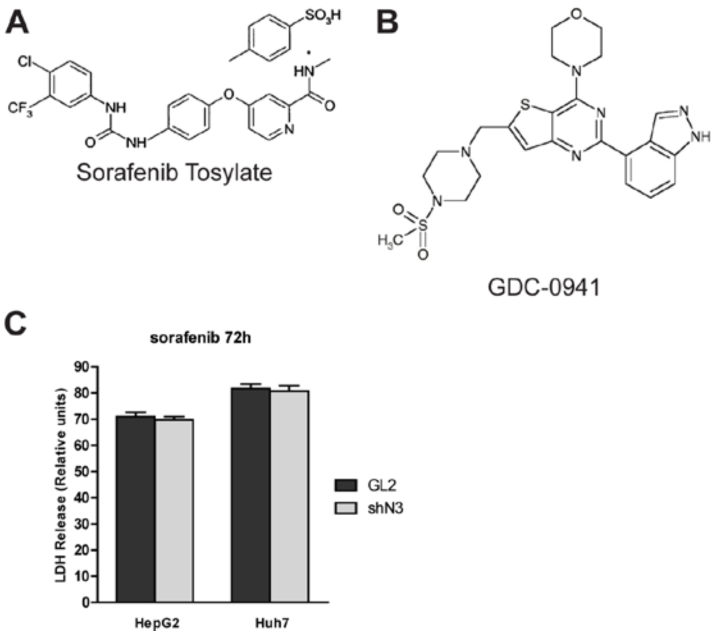

Supp. Figure 1: LDH levels in response to sorafenib treatment.

(A) Structure of sorafenib tosylate. (B) Structure of kinase inhibitor GDC-0941.

(C) Seventy-two hours post sorafenib treatment levels of LDH were measured in the supernatants of Notch3 depleted cells (shN3) and control cells (GL2). Columns are the means of two independent experiments (+/- S.E.).

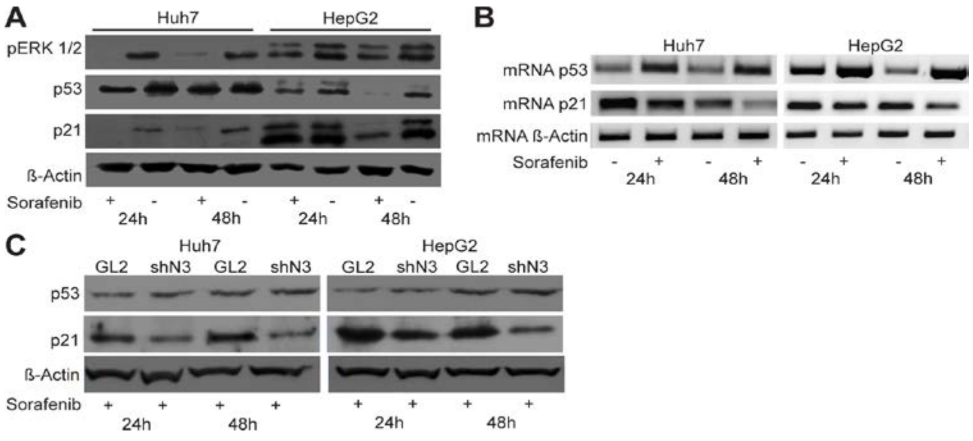

Supp. Figure 2: Effects of sorafenib on specific protein and gene expression.

Huh7 and HepG2 cells were treated with 4 µM of sorafenib for 24 h and 48 h. (**A)** Western blot of pERK1/2, p53 and p21 relative to a β actin protein reference control. Sorafenib treatment down-

regulates the expression of p53 and p21 in both cell lines. (**B**) Semi-quantitative RT-PCR expression analysis of p21 and p53 genes. (**C**) Huh7 and HepG2 cells, stably transduced with either GL2 or Notch3 shRNA retrovirus were treated with 4 μM of sorafenib for 24 h and 48 h and the expression levels of p53 and p21 were analyzed by western blot. GL2: negative control shRNA; shN3; Notch3 shRNA.

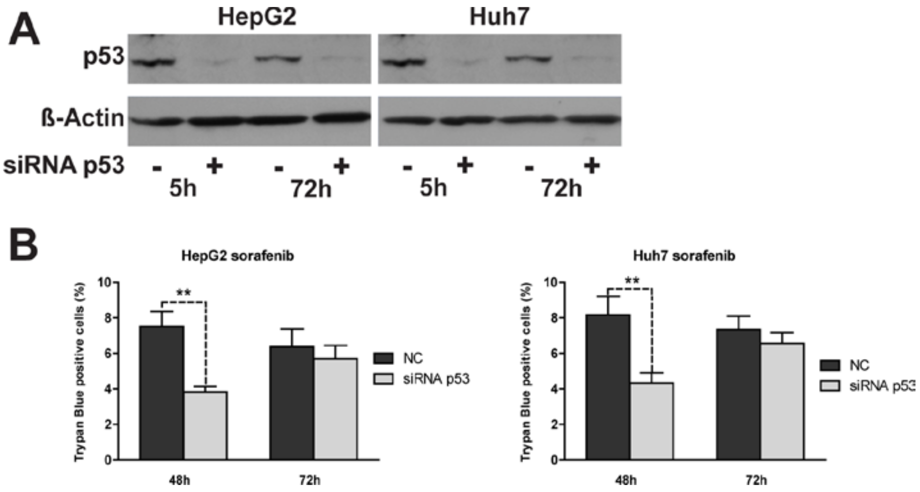

Supp. Figure 3: Effect of p53 silencing on sorafenib sensitivity. (A) HepG2 and Huh7 cells were transfected with p53 siRNAs or with scrambled siRNAs (NC) and p53 knockdown was evaluated 5 h and 72 h post-transfection by western blotting. (B) The effect of p53 silencing on sorafenib (4 μM) induced cell death was assessed by trypan blue uptake as the means of three independent experiments (+/- S.E.) Sorafenib treatment was performed 5h post p53siRNAs transfection. P value, evaluated by two tailed student's t test, was $P<0.01$ (**) and $p=0.06$ after 48 h and 72 h of sorafenib exposure respectively.
